# Comparative effects of saline, streptomycin, and ozone nasal irrigation on *Staphylococcus aureus-*induced experimental rhinosinusitis: microbiological, histopathological, and biofilm analyses

**DOI:** 10.1016/j.bjorl.2026.101916

**Published:** 2026-10-02

**Authors:** Gökhan Tüzemen, Nazmiye Ülkü Tüzemen, Zehra Avcı Küpeli, Cüneyt Özakın

**Affiliations:** a Ministry of Health, Bursa City Hospital, Department of Otorhinolaryngology-Head and Neck Surgery, Bursa, Turkey; b Bursa Uludag University, Faculty of Medicine, Department of Medical Microbiology, Bursa, Turkey; c Bursa Uludag University, Faculty of Veterinary Medicine, Department of Pathology, Bursa, Turkey

**Keywords:** Biofilm, Streptomycin, Ozone, Irrigation, Rat

## Abstract

**Objective:**

Rhinosinusitis is characterized by inflammation of the nasal mucosa and paranasal sinuses. Nasal irrigation is widely used as an adjunctive treatment for sinonasal diseases. The aim of this study was to evaluate whether nasal irrigation with streptomycin or ozone provides additional benefit compared with saline irrigation alone.

**Methods:**

We used 22 female Sprague–Dawley rats to induce bacterial rhinosinusitis. Group 1 was not irrigated, group 2 was irrigated with saline, group 3 with streptomycin, and group 4 with ozone solution. After the treatment, sinus mucosa samples were evaluated using microbiological methods to measure bacterial load. The histopathological inflammation rate was determined, and biofilm formation was assessed semi-quantitatively using scanning electron microscopy.

**Results:**

After irrigation, a significant reduction in bacterial counts was observed in all groups. In the streptomycin group, viable bacteria in the lumen were largely reduced; however, bacteria that adhered to the mucosa remained. Although the streptomycin group showed the lowest average scores for both biofilm and inflammation, these differences were not statistically significant. Overall, there was no statistically significant difference between the groups.

**Conclusions:**

This experimental study explored biofilm formation in an acute bacterial rhinosinusitis model. Irrigation procedures were associated with a reduction in bacterial load, but no statistically significant differences were observed between groups in terms of histopathological findings or biofilm formation. These findings should be interpreted cautiously, and further studies with larger sample sizes are required to clarify their potential clinical relevance.

**Levels of evidence:**

Not applicable (preclinical animal model).

## Highlights

• The most significant decrease in bacterial counts was observed in streptomycin group.
• Streptomycin group had the lowest pathological and SEM scores.
• This is the first study of using streptomycin irrigation in sinusitis.

## Introduction

Rhinitis is inflammation or dysfunction of the nasal mucosa, causing nasal symptoms.^1^ Rhinosinusitis is the inflammation of the nasal mucosal membrane and one or more paranasal sinuses, with a high prevalence.^2^ Acute Rhinosinusitis (ARS) can result from viral, bacterial, or fungal infections, environmental irritants, or allergies. Acute Bacterial Rhinosinusitis (ABRS) is typically caused by a secondary bacterial infection in the sinus.^3^ Sinus mucosal edema, obstruction of sinus ostia, and diminished mucociliary function are critical to rhinosinusitis pathophysiology, leading to stagnant secretions and bacterial growth. In adults with suspected acute maxillary sinusitis post-viral infection, about fifty percent had pus or mucopus, and one-third had bacterial pathogens. Common bacteria include *Streptococcus pneumoniae*, *Haemophilus influenzae*, and *Moraxella catarrhalis*.^4^ *Staphylococcus aureus* is a significant opportunistic pathogen frequently present in the sinonasal microenvironment.^5^

Biofilm formation is a complex process comprising several stages. It begins with the attachment of free-floating microorganisms to a surface, followed by microcolony formation, maturation, and ultimately, dispersion. This final phase allows the biofilm to spread and colonize new surfaces. Biofilms are thought to account for approximately 60% of all human bacterial infections, 65% of hospital-acquired infections, and around 80% of chronic infections. Biofilms are essential in the emergence of antimicrobial resistance and the development of chronic infections. They modulate host immune responses, contaminate medical devices, and persist in hospital effluents, acting as reservoirs for drug-resistant genes.^6^

Nasal irrigation is a traditional method for cleansing the upper respiratory tract, originating from ancient Ayurveda. It is used to alleviate symptoms of rhinosinusitis and allergies and was introduced to Western medicine in the early 19th century. Nasal irrigation is effective as a standalone treatment or in conjunction with other therapies, particularly in addressing upper respiratory infections in children. Otorhinolaryngologists and pediatricians support its use for symptom relief and as an adjunct to pharmacological treatments.^7^ The first study on using streptomycin locally in treating sinusitis was published in 1948.^8^ Otolaryngologists in some clinical settings have recommended local nasal irrigation with streptomycin saline for persistent sinusitis. This formula, prepared as a magistral formula by the pharmacy, has shown positive clinical responses. Streptomycin has been found to inhibit biofilm formation and destroy mature biofilms.^9^^,^^10^ Ozone is a potent oxidant with three oxygen atoms. It can prevent bacterial biofilm formation and has potent bactericidal activity.^11^^,^^12^

In our previous study, we evaluated the antibiofilm efficacy of streptomycin and ozone solutions against strains of *S. aureus* and *Pseudomonas aeruginosa* that colonize the respiratory tracts of cystic fibrosis patients. We demonstrated that both ozone and streptomycin solutions exhibited significant inhibitory effects. However, ozone showed no inhibitory action on mature *P. aeruginosa* biofilms. Consequently, we selected the *S. aureus* strain with the highest biofilm activity for this study. We have designed a model of rat ABRS using *S. aureus*. We inoculated bacteria into the nasal cavity of all rats and then performed nasal irrigation using saline, saline with streptomycin, and ozone solution in experimental groups. The objective of this study was to assess the efficacy of irrigation with streptomycin solution or ozone in comparison to saline irrigation for the treatment of *S. aureus* induced ABRS and the associated *S. aureus* biofilm.

## Methods

### Bacterial preparation

In our previous study, *S. aureus* was isolated from the sputum sample of a patient with cystic fibrosis. This isolate demonstrated the strongest biofilm-forming capacity, which was confirmed by the microtiter plate assay based on its high Optical Density (OD) values.^13^ The clinical *S. aureus* isolate used in this study was obtained with written informed consent, and ethical approval was granted by our University, Faculty of Medicine Ethics Committee (no: 2022-13/21). The night before the experiment, a loop of the *S. aureus* strain was inoculated in a 5 mL Tryptic Soy Broth (BD, USA) medium, supplemented with 1% glucose (TSBg), and then incubated overnight at 37 °C in an orbital shaker at 50 rpm (Zhicheng, Shanghai, China). Then, the bacterial suspension was diluted with new TSBg to achieve a concentration of approximately 0.5 McFarland (1 × 10^8^ colony-forming units [CFU]/mL) and kept at 4 °C until further use.^14^

### Irrigation solutions

In our study, we utilized three different types of irrigation solutions, each with an application of 3 mL of liquid. The first solution was a 0.9% saline. For the second solution, we added streptomycin to the 0.9% saline. Fineberg^8^ used 2 mg/mL of streptomycin in saline to irrigate the maxillary sinus. He used a catheter to insert the solution into the sinus and then irrigated it. We used 8 mg/mL of streptomycin for nasal irrigation in saline because a small volume can pass into the maxillary sinus with reasonable pressure. The third solution was an ozone solution. The ozone formulation used in this study was a commercially available product (Geoxyn Nanotech Geoxynoral 1600), designed as a nasal-oral spray. The formulation contains ozone-coated nanomolecules prepared at a concentration of 1600 ppm (approximately 1.5 mg/mL) and is based on an ozonated nanoliposome structure decorated with hyaluronic acid (Patent no: PCT/TR2022/050177). The product was used in its ready-to-use commercial form without further dilution or modification. According to the manufacturer’s specifications, the nanoliposome-based formulation provides enhanced stability of the ozonated components compared with conventional aqueous ozone solutions. The solution was stored under the recommended conditions and freshly applied during the irrigation procedures. It should be noted that this formulation is not a pure aqueous ozone solution but rather a decorated ozonated nanoliposome formulation, which is designed to improve stability and mucosal compatibility. Previous evaluations of biocompatibility and toxicity for skin, oral, nasal, and ocular exposure have reported no evidence of toxicity associated with the product.^15^

### Rats and experimental design

Ethical approval was obtained from the Experimental Animal Breeding and Research Unit of the Faculty of Medicine at our University (Approval ID: 2022-10/04). The rats utilized in this study were acquired from the same unit. All methods were carried out in accordance with relevant guidelines and regulations. Our study was designed following the recommendations of Dogan et al.^16^ to use the minimum number of animals possible. Sample size was estimated using the resource equation method, which is commonly applied in exploratory animal studies when prior variance estimates are not available. According to this approach, the error degrees of freedom (E) should fall between 10 and 20, where *E = N – G* (*N* = total number of animals; *G* = number of groups). Accordingly, the total number of animals is determined using the inequality *G + 10 ≤ N ≤ G + 20*. To estimate the number of animals per group, the following formulas were applied: Minimum animals per group = (G + 10) / G; Maximum animals per group = (G + 20) / G.

For four groups, this calculation yielded a minimum of four and a maximum of six animals per group. Based on this estimation, six animals were initially planned for each group. However, following the recommendation of the institutional animal ethics committee and in accordance with the principles of reduction within the 3R framework, the control group size was reduced to the minimum number (n = 4), while the experimental groups included six animals each.

To further evaluate the statistical robustness of the design, a post-hoc power analysis was performed using G*Power software (version 3.1.9.7). Assuming a one-way ANOVA design with four groups, an effect size of *f* = 0.40 and α = 0.05, the achieved statistical power with a total sample size of 22 animals was calculated as 26%. An a priori power analysis indicated that a total sample size of 76-animals would be required to achieve a statistical power of 80% under the same assumptions.

We used 22 female Sprague-Dawley (SD) rats aged between 6–8 weeks and weighing 230 ± 10 g. All experiments were conducted in compliance with the ARRIVE guidelines.^17^ Rats were maintained under standard conditions throughout the experiment, and no systemic antibiotics were given before or during the study.

The rats were randomly divided into four groups for the study. Group 1 was the control group (four rats), while Groups 2, 3, and 4 were the experimental groups (six rats in each group). Group 1 was not irrigated, Group 2 was irrigated with saline, Group 3 with streptomycin in saline, and Group 4 with ozone solution. The rats were individually coded for each group in alphabetical order. Fig. 1 illustrates the interventions applied to each study group. This study was conducted in three phases. In the first phase, Merocel sponges (Medtronic Xomed, Inc., Jacksonville, FL, USA) were inserted to block one nostril, and nasal bacteria (*S. aureus*) were inoculated. The second phase involved the irrigation of the experimental groups. Finally, in the third phase, data were evaluated after the rats were sacrificed (decapitated).

**Fig. 1:**
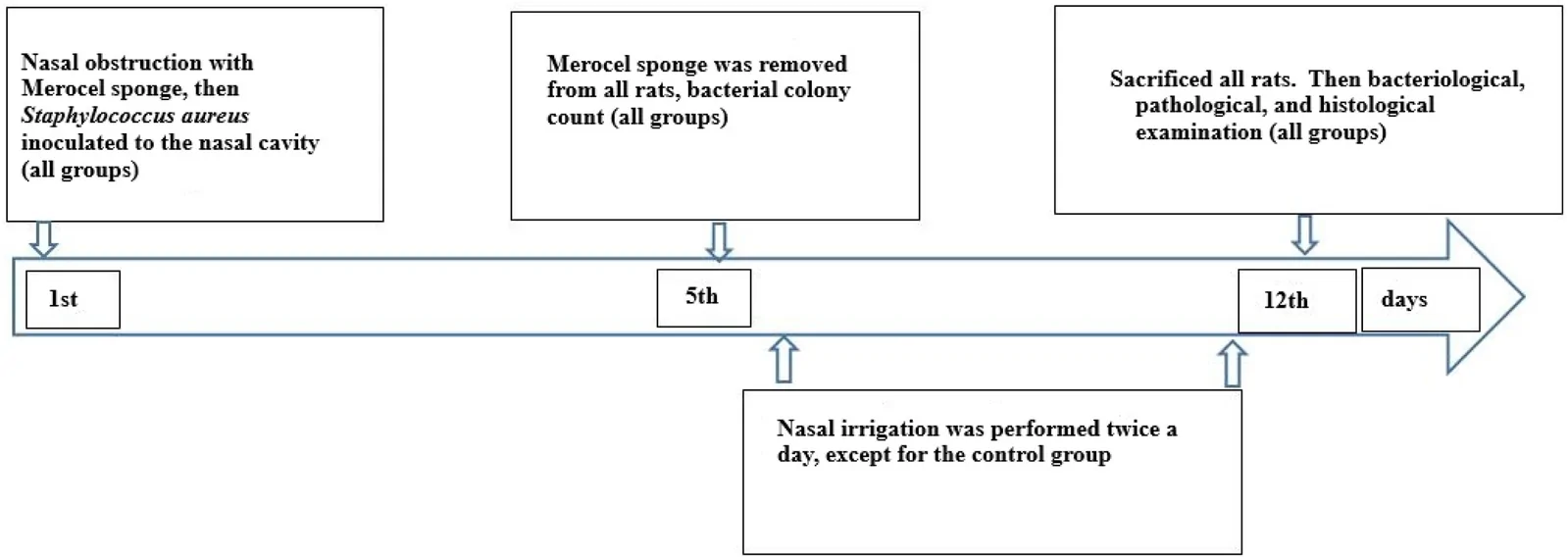
The timeline of the experiment.

This study was conducted on rats to induce an inflammatory process in the nasal cavity, simulating ABRS. The rats were placed in a chamber where they were exposed to air containing 2%–8% sevoflurane. To induce rhinosinusitis, 0.2 mL of a 0.5 McFarland suspension of *S. aureus* was inoculated into the right nasal cavity (see Fig. 2a). Following the inoculation, a Merocel sponge, measuring 1.5 × 1.5 × 10 mm, was inserted into the same nasal cavity using sterile bayonet forceps (refer to Fig. 2b).^18^ Purulent nasal secretions were observed in the nasal cavities of the rats on the fifth day (Fig. 2c), at which point the Merocel sponge was removed from all groups. Three experimental groups (Groups 2, 3, and 4) received rinses of room-temperature irrigation solutions twice daily (3 milliliters) for one week, administered at 9:00 a.m. and 4:00 p.m. via 20-gauge intravenous cannulae inserted approximately 1 cm into the nasal cavity (Fig. 2d). Irrigation was performed under normal manual pressure, and the instilled solution reached the maxillary sinus in all animals. After one week, the rats were euthanized by decapitation, and nasal lavages were cultured for bacteriological analysis. The nasal cavities of rats are relatively small, leading to inflammation affecting the entire region. Therefore, sampling was conducted from the entire nasal cavity. The sinonasal mucosa was dissected, with each sample cut into three parts for different experiments (Fig. 2e). These experiments included bacteriological culture, histopathological examination, and Scanning Electron Microscopy (SEM) specimen preparation. All procedures were conducted following the principles of aseptic technique by an experienced otorhinolaryngology surgeon.

**Fig. 2:**
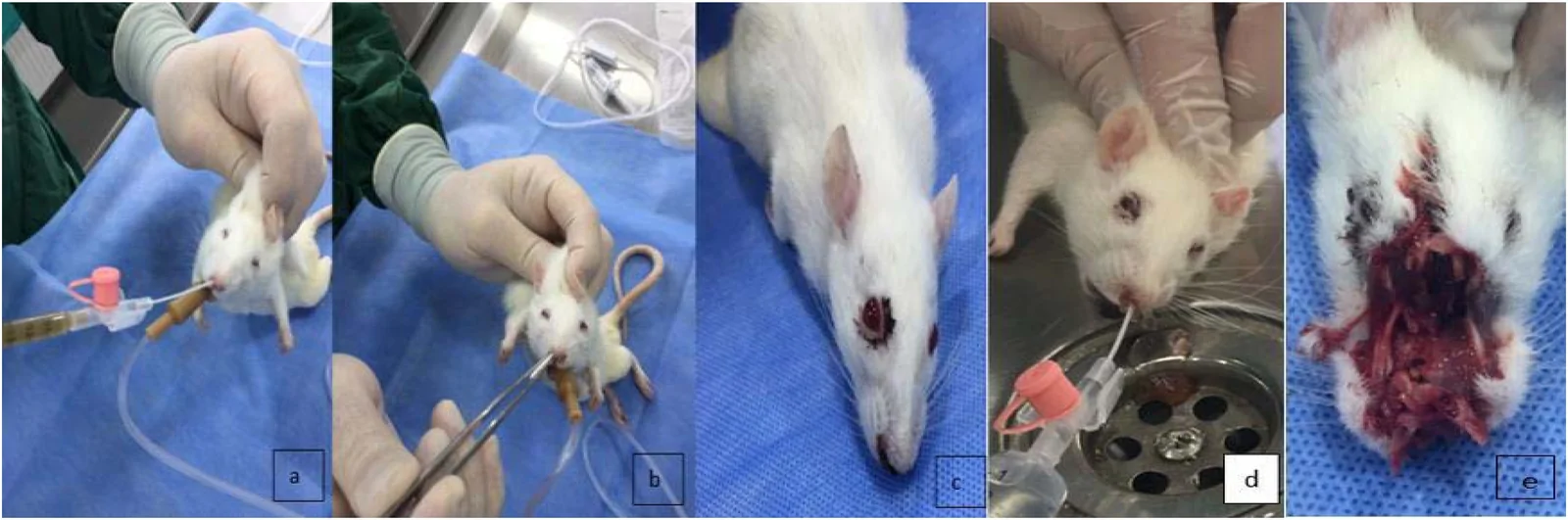
(a) Bacterial inoculation, (b) Inserting Merocel sponge, (c) Purulent nasal and ocular secretion after the induced rhinosinusitis, (d) Nasal irrigation, (e) Surgical dissection of nasal tissues.

### Bacterial culture and strain identification

Nasal lavage samples were collected before and after the irrigation treatment. The samples were diluted at a ratio of 1:100, and then 10 μL of the diluted lavage fluid was placed on a 5% sheep blood agar plate (BD, USA). The agar plate was incubated at 37 °C overnight.^19^ On the fifth day, the Merocel sponge was removed and cultured. On the twelfth day, the nasal mucosa tissue was dissected and cultured.

### Histopathological specimen preparation

Mucosal specimens were examined in the Pathology Department of the Faculty of Veterinary Medicine. The investigator was blinded to the treatment type when assessing the histological inflammation. The specimens were fixed in 10% buffered formalin for 48 hours, then embedded in paraffin. Microtomy was performed, with each slice having a maximum thickness of 4 μm. Finally, the slices were stained with Hematoxylin-Eosin (H&E). The samples were classified based on inflammation criteria as follows: 0 ‒ No inflammation; 1 ‒ Mild inflammation; 2 ‒ Moderate inflammation; 3 ‒ Intense inflammation; and 4 ‒ Severe inflammation with ulceration.^20^

### SEM specimen preparation

A Scanning Electron Microscopic (SEM) examination was conducted in the Central Research Laboratory of the university. Tissue specimens were processed according to standard SEM preparation protocols and examined under a scanning electron microscope to evaluate mucosal surface morphology and bacterial biofilm structures. Biofilm formation was assessed using a semi-quantitative scoring system based on SEM images. The scoring system was applied to evaluate the extent of biofilm coverage on the mucosal surface. The investigator who evaluated the SEM images was blinded to the treatment groups during the assessment. All tissue samples were initially placed in 2.5% glutaraldehyde, prepared in 0.1 M phosphate buffer at pH 7.4, for 24 -hs at 4 °C as a prefixation step. After this, the samples were rinsed twice with 0.1 M phosphate buffer (pH 7.4) and then subjected to postfixation using 1% osmium tetroxide for 1 -h at room temperature. The specimens were rinsed again with distilled water.

Next, the specimens were dehydrated using graduated concentrations of ethyl alcohol (30%, 50%, 70%, 90%, and 96%) for 15-minutes each, followed by immersion in absolute alcohol for 30-minutes. The specimens were then dried using the Polaron Critical Point Dryer (VG.Microtech, East Sussex, UK). For mounting and coating, carbon conductive paint was applied, followed by gold coating using the Polaron Sputter Coater.

Each specimen was examined using a JEOL scanning electron microscope (JEOL JSM-5600LV). A sample was considered to have a biofilm if it contained bacterial-sized and shaped objects, an amorphous material consistent with glycocalyx surrounding the bacteria, and surface binding. Multiple areas of each sample were systematically scanned.

For the quantitative analysis of biofilm formation, nine fields of vision were randomly selected for each specimen. The presence of biofilm was classified according to the following criteria^14^: 0: No biofilm formation; 1: Biofilm formation present in 1–3 regions; 2: Biofilm formation present in 4–6 regions; 3: Biofilm formation present in 7–9 regions.

### Statistical analysis

All statistical analyses were performed using R software (version 4.5.2; R Foundation for Statistical Computing). Histopathology scores, SEM scores, and bacterial load (CFU/mL) values were analyzed using non-parametric tests, as these variables represent ordinal or non-normally distributed data by design. Comparisons among the four groups were conducted using the Kruskal-Wallis test, followed by Dunn’s post-hoc test with Benjamini-Hochberg correction for multiple comparisons. Graphical representations were generated using the ggplot2 package. A significance level of p < 0.05 was applied for all analyses.

## Results

The *S. aureus* strain showed a methicillin-resistant and inducible MLSB phenotype, displaying resistance to erythromycin, and clindamycin, whereas it retained susceptibility to linezolid, vancomycin, teicoplanin, tetracycline, fosfomycin, and trimethoprim-sulfamethoxazole. In terms of aminoglycoside susceptibility, the isolate showed susceptibility to amikacin, while exhibiting resistance to gentamicin.

During this study, we observed that all rats developed sinusitis, confirmed by the presence of mucopurulent secretions in each rat. The irrigation procedures were conducted using the smallest intravenous cannulae (20-gauge). Four rats, identified as 3 F, 4 F, 2B, and 2C, died during the study. Specifically, 3 F died on the 4th-day of the irrigation, 4 F on the 6th-day, and both 2B and 2C on the 7th-day, just before the second irrigation of the final day. We collected nasal tissue from these rats on the day they died and performed bacteriological, pathological, and electron microscopy examinations. However, no nasal lavage was conducted on these rats prior to their sacrifice, and only tissue culture was performed for bacteriological analysis.

### Bacteriological analysis

*Staphylococcus aureus* was detected in cultures obtained from all rats. Animals that died during the experiment were excluded only from the statistical analysis of bacterial load, as nasal lavage samples could not be obtained prior to death. Tissue samples from these animals were nevertheless included in the histopathological and SEM analyses. In the first group, which did not receive any irrigation, the bacterial count decreased after removal of the occlusive Merocel sponge. Overall, bacterial counts decreased in all groups following the irrigation procedures.

A total of four experimental groups were compared in terms of nasal lavage bacterial load (CFU/mL) on the 5th and 12th-days. The distributions of CFU values for each group and the pairwise group comparisons at each time point are presented in Fig. 3 and Table 1. On Day-5, Group 1 showed the highest bacterial load, followed by Groups 2, 4, and 3. Pairwise comparisons using the Wilcoxon rank-sum test revealed no statistically significant differences between Group 1 and Group 2 (p = 0.39), Group 1 and Group 3 (p = 1), or Group 1 and Group 4 (p = 0.71). On Day-12, the overall CFU levels decreased across all groups. Group 1 remained higher than the other groups, while Groups 2, 3, and 4 exhibited lower bacterial counts. Pairwise comparisons demonstrated that Group 1 differed significantly from Group 2 (p = 0.029), Group 1 from Group 3 (p = 0.015), and Group 1 from Group 4 (p = 0.018). Within-group longitudinal changes (Day-5 vs. Day-12) were assessed using paired Wilcoxon signed-rank tests. Although all groups showed a numerical decrease from Day-5 to Day-12, the reductions were not statistically significant: Group 1 (p = 0.125), Group 2 (p = 0.125), Group 3 (p = 0.0579), and Group 4 (p = 0.0625). These p-values are shown beneath the corresponding panel in Fig. 3.

**Fig. 3:**
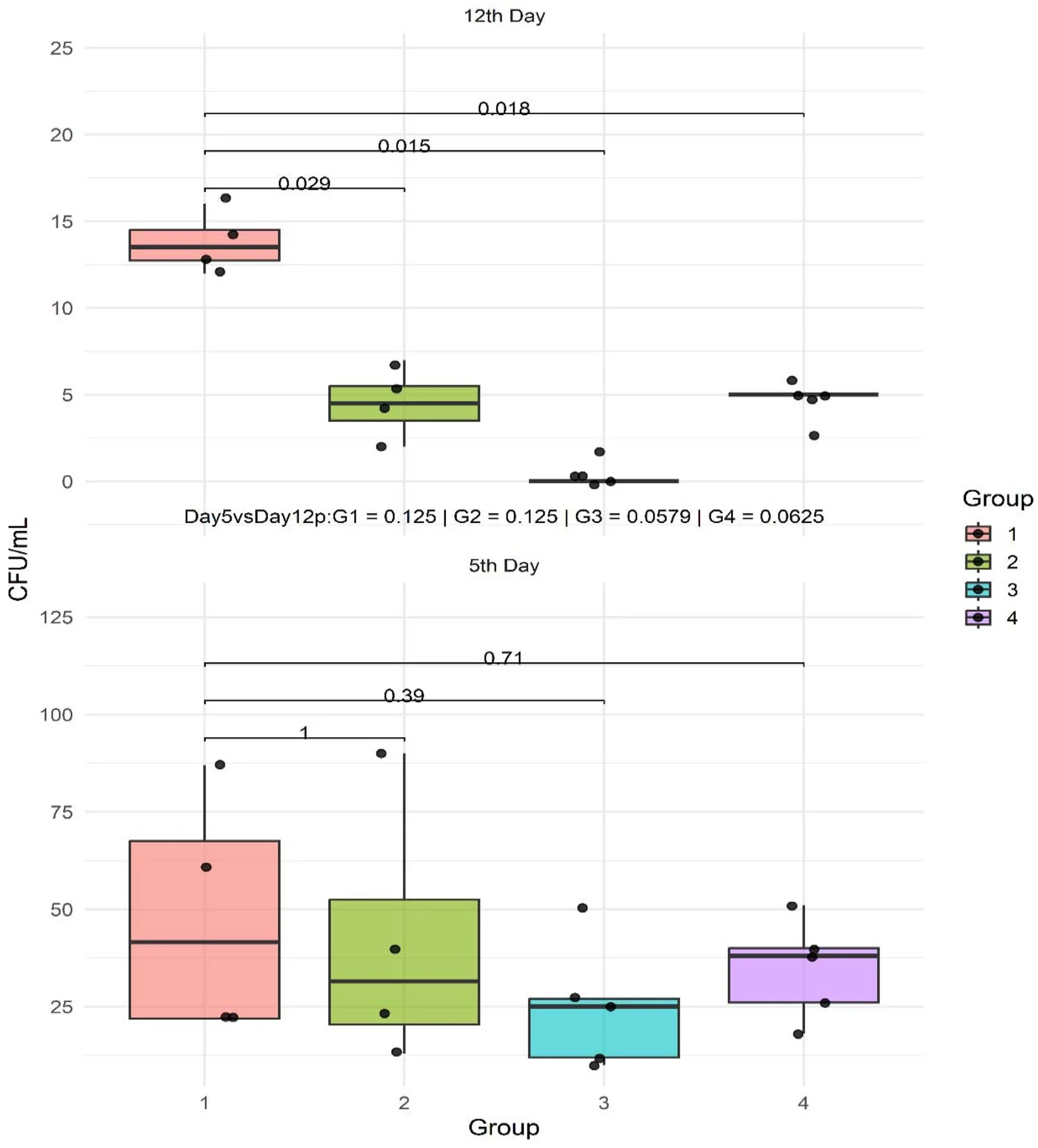
CFU levels measured in nasal lavage samples on Day-5 and Day-12 across the four groups. Boxplots show distributions with individual data points. Between-group differences were tested with the Wilcoxon rank-sum test (brackets), and within-group Day-5 – Day-12 changes with paired Wilcoxon tests (values shown below).

**Table 1:** Colony-forming unit (CFU/mL) counts obtained from nasal lavage samples of rats on Day-5 and Day-12 across four experimental groups.

Rat code	Nasal Lavage (CFU/mL) (5-day)	Nasal Lavage (CFU/mL) (12-day sacrification)
1A	61	13
1B	87	12
1C	22	14
1D	22	16
2A	90	2
2B	36	ex
2C	37	ex
2D	13	5
2E	40	7
2F	23	4
3A	50	0
3B	12	2
3C	27	0
3D	10	0
3E	25	0
3F	23	ex
4A	40	3
4B	51	6
4C	38	5
4D	26	5
4E	18	5
4F	33	ex

Overall, the results indicate that while all groups demonstrated a reduction in bacterial burden over time, the decrease did not reach statistical significance within groups. However, on Day-12, Group 1 maintained significantly higher CFU levels than Groups 2–4.

### Histopathological analysis

Histopathological evaluation was performed on tissue samples obtained from all rats, including those that died during the experimental period, as tissue samples were collected immediately after death. Lesions were identified in the pseudostratified columnar epithelium of the initial group, which exhibited disorganization and ulceration resulting from cellular shedding. Moderate connective tissue formation and diffuse infiltration of inflammatory cells were observed in one area of the submucosa. In groups 2 and 4, localized bleeding areas and clusters of bacteria were documented in the lamina propria. It is important to note that the inflammatory scores among all groups did not demonstrate a significant difference (p = 0.452). The specifics of the inflammation scoring are illustrated in Fig. 4, while the H&E-stained sections of nasal tissues corresponding to each score are displayed in Fig. 5.

**Fig. 4:**
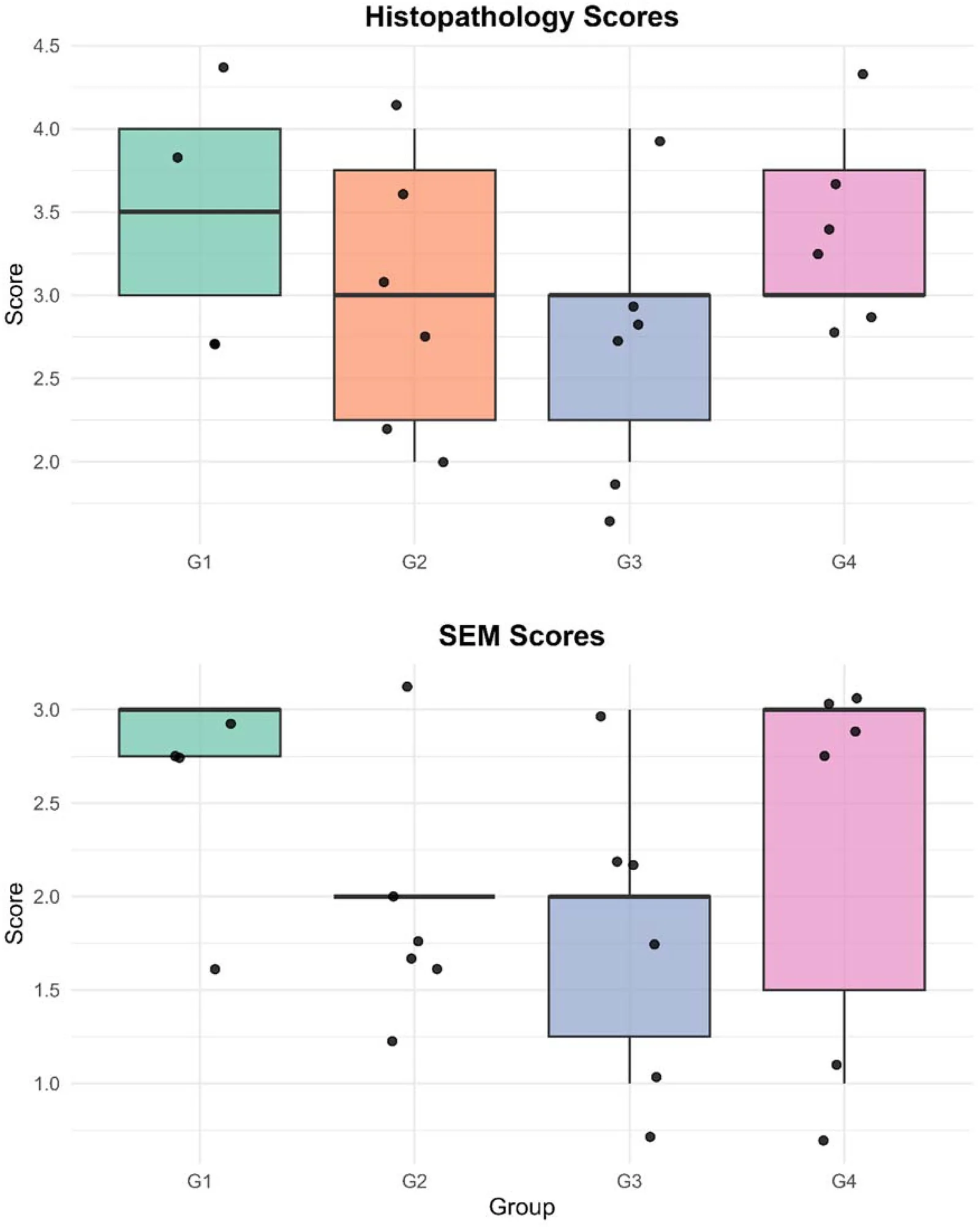
Histopathology and Scanning Electron Microscopy (SEM) scores of nasal tissue in the four study groups.

**Fig. 5:**
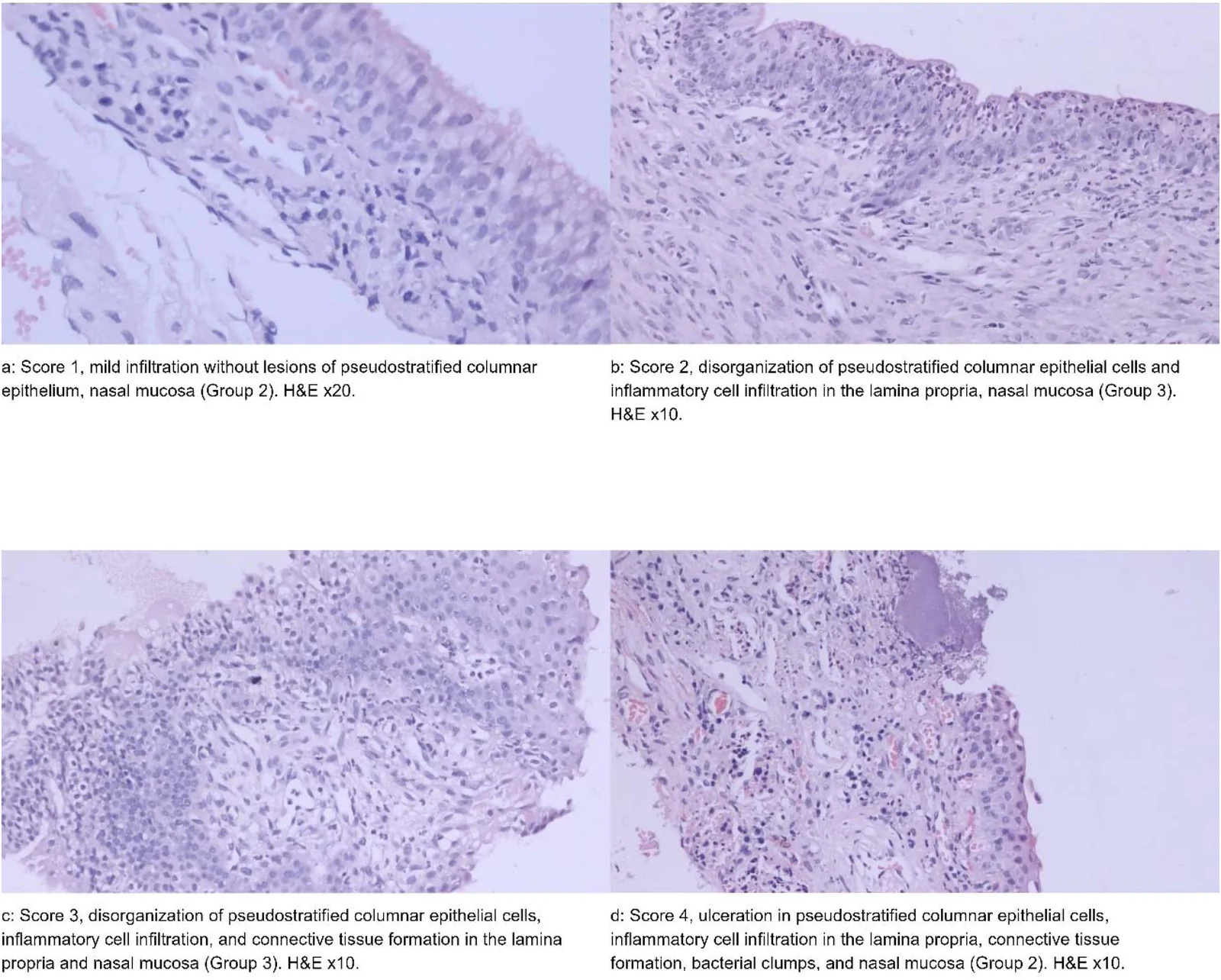
Hematoxylin-Eosin stained images for each histopathological score category.

### SEM analysis

SEM analysis included tissue samples from all rats, as specimens were collected immediately after death in animals that did not survive the full experimental period. Each sample was carefully examined, and data were collected from nine different regions based on the presence or absence of biofilm. Regions showing dense aggregations of microorganisms embedded in the extracellular matrix were classified as biofilmpositive. According to the SEM data, there was no statistically significant difference in grades among the groups (p = 0.248). The scores for biofilm formation observed through SEM are presented in Fig. 4, and SEM images of biofilm-positive samples can be found in Fig. 6.

**Fig. 6:**
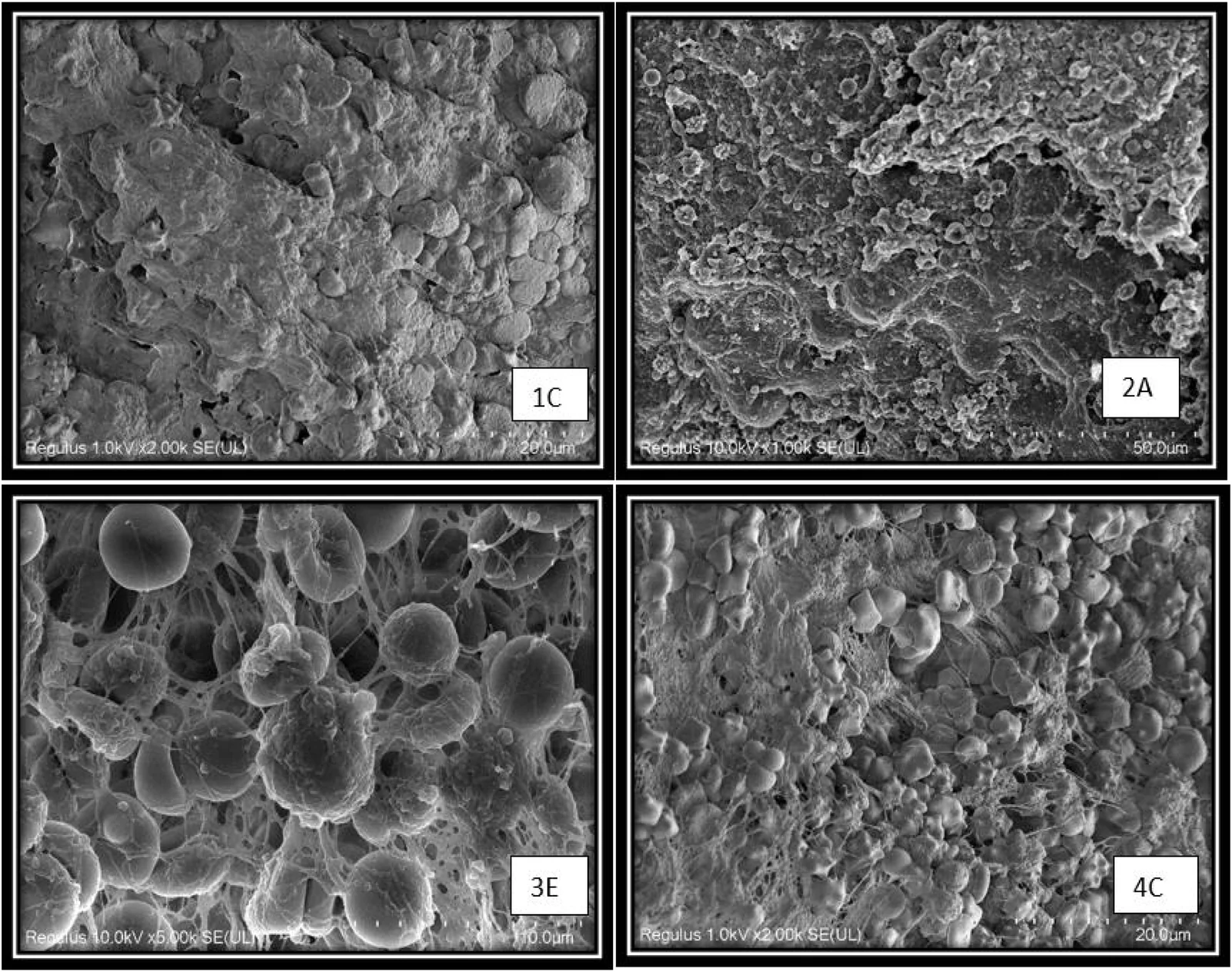
Images of the nasal mucosa from each group were taken using scanning electron microscopy (The numbers represent the rat group, while the letters denote the individual rat's code within that group).

## Discussion

Rhinosinusitis occurs when the mucosal lining of the nasal cavity and sinuses becomes inflamed. Since sinus inflammation rarely happens without involving the nasal mucosa, it is more accurate to refer to this condition as rhinosinusitis rather than sinusitis.^4^ The inflammation of the nasal and paranasal mucosa is often caused by respiratory viruses and bacteria.^21^ In fact, half of the patients with acute maxillary sinusitis following a viral respiratory infection show pus or mucopus in their sinus aspirate, while one-third have bacterial pathogens identified in culture.^4^ Although *Staphylococcus aureus* is less frequently a cause, it poses a higher risk of complications related to ABRS.^21^ Both aerobic and anaerobic microorganisms can form biofilms that trigger the release of inflammatory molecules during rhinosinusitis.^22^ The duration of each stage in biofilm formation can range from nanoseconds to weeks, depending on factors such as the size of the inoculum and the mechanism of colonization. In some cases, biofilm formation can be detected in under 60-minutes.^23^

If the patient's condition does not improve within seven to ten days or worsens at any time, consider starting antibiotic therapy.^4^ An analysis of the literature indicates that in the absence of antibiotic treatment, 46% of individuals diagnosed with rhinosinusitis, regardless of radiographic confirmation, experienced resolution of symptoms after one week, while 64% achieved resolution after fourteen days.^24^ The utilization of nasal saline sprays or irrigations may also contribute to relief from symptoms associated with ARS.^21^ A study found that normal saline irrigation improved symptoms, quality of life, and nasal peak expiratory flow rates in pediatric patients.^25^

Monitoring biofilm production is not commonly practiced in microbiological analysis, but identifying biofilm-forming bacteria like *S. aureus* and *Pseudomonas aeruginosa* is vital for clinicians. Targeted antimicrobial therapy based on culture results can reduce biofilm formation, prevent chronic inflammation, and improve patients' quality of life.^26^ In our previous study, we investigated the effectiveness of streptomycin and ozone solutions in preventing biofilm formation by *S. aureus* and *P. aeruginosa* strains isolated from the respiratory tracts of cystic fibrosis patients. Our results indicated that both treatments significantly inhibited biofilm growth; however, ozone was ineffective against mature biofilms of *P. aeruginosa*. Consequently, we chose the *S. aureus* strain with the highest biofilm activity for this animal model study.

Unlike conventional aqueous ozone solutions, which are characterized by rapid degradation, the nanoliposome-based formulation used in the present study is designed to enhance the stability of ozonated molecules and improve mucosal compatibility.

Nasal saline irrigation is widely recognized as an effective and safe treatment for sinonasal diseases in adults, thanks to its cost efficiency.^27^ Sinonasal anti-infective irrigations are a promising option for treating sinusitis, especially in cases that do not respond to standard therapies. While not a first-line treatment, they may help patients with inadequate results from conventional methods.^28^ In our study, we observed a statistically significant reduction in bacterial load across all irrigation groups when compared to the control group. This finding is likely attributable to the mechanical cleansing effect imparted by the irrigation process, independent of the composition of the fluid utilized.

Various animal models have been employed in the literature to simulate sinonasal inflammation.^29^ Following the 3R principle, we aimed to use the minimum number of animals necessary for our study.^30^ The relatively low statistical power reflects the small sample size inherent to exploratory animal studies. Increasing the statistical power to the commonly accepted level of 80% would require approximately 76 animals, which would conflict with ethical considerations and the principles of animal reduction (3R principle). Therefore, the resource equation method was used to determine a feasible and ethically acceptable sample size. Different methods have been utilized to induce bacterial sinus infections in these models.^14^^,^^18^^,^^19^^,^^20^ We conducted unilateral nasal obstruction induction because there was no significant difference in the bacterial and histopathological characteristics between the two sides of the nasal tissue.^31^

Streptomycin has a low incidence of resistance but faces limitations in use due to dose-related toxicity, patient adherence, and the need for parenteral administration. Its bioactivity diminishes in the acidic stomach, and it has poor intestinal permeability due to its hydrophilic nature and high molecular weight. A study demonstrated that using lipid nanoparticles for streptomycin delivery significantly increased its concentrations in the brain and blood of mice, achieving levels 3.15- and 11.0-times higher than free streptomycin, respectively, in cases of tuberculous meningitis.^32^ A study assessed gentamicin administration in ten healthy dogs for upper respiratory disease. Three protocols were tested: 10-min nebulization, 3-min nebulization, and a drop protocol. The drop protocol resulted in the highest gentamicin concentration in nasal lavage fluid (137.00 μg/mL), significantly higher than the nebulization protocols. Serum levels were higher in the 10-min nebulization but often undetectable in the drop protocol. All methods achieved effective concentration levels without toxic serum levels.^33^ In a study investigating the use of irrigation with the aminoglycoside tobramycin in patients diagnosed with cystic fibrosis, it was observed that only 2 out of 10 patients exhibited detectable serum levels of tobramycin. These levels were notably low and remained well within the acceptable limits, far below the threshold for toxicity.^34^ A limitation of this study is the absence of serum streptomycin measurements, which precludes a definitive assessment of potential low-level systemic absorption.

During our study, four rats died before completion of the experiment. These fatalities may have resulted from several factors, including stress associated with the experimental conditions or the development of a severe systemic infection such as sepsis. Although the animals were monitored regularly and nasal irrigation was performed twice daily, no obvious reduction in movement or clinical deterioration was observed prior to death. The absence of blood culture analysis from the deceased animals prevented confirmation of bacteremia or sepsis, representing a limitation of this study.

Tissue samples from these animals were collected immediately after death and were therefore included in the histopathological and SEM evaluations. However, nasal lavage samples could not be obtained prior to death, and these animals were excluded from the quantitative bacterial load analysis. Consequently, the microbiological analysis was performed with a reduced sample size, which may have decreased the statistical power to detect subtle differences between groups and may have introduced potential bias in the microbiological findings.

Another limitation of this study relates to the method used for biofilm evaluation. Biofilm formation was assessed using a semi-quantitative scoring system based on scanning electron microscopy images. Although SEM allows detailed visualization of bacterial adherence and biofilm architecture on the mucosal surface, it does not provide fully quantitative measurements of biofilm biomass. More advanced techniques, such as confocal laser scanning microscopy combined with fluorescent staining, may allow more precise quantification of biofilm structure and thickness. The absence of such quantitative methods in the present study may have limited the ability to detect subtle differences in biofilm formation between the experimental groups.

All rats exhibited positive results for *S. aureus* during the microbiological analyses. Following irrigation, bacterial counts diminished across all treatment groups. Within the streptomycin group, only one rat presented with bacteria in the second lavage, although the colony count had decreased. One rat died before the second lavage procedure. No bacterial growth was observed in the remaining rats of the streptomycin group. Since streptomycin is a bactericidal antibiotic, the absence of bacterial growth in the second lavages likely resulted from the twice-daily irrigation regimen over seven days. Streptomycin effectively eliminates viable bacteria in the sinus lumen, as corroborated by similar research wherein rabbits were irrigated with tobramycin in saline, leading to the eradication of viable *P. aeruginosa* from the sinus lumen; however, residual bacteria remained adhered to the sinus mucosa.^35^ Despite the bactericidal attributes of ozone, it did not successfully eliminate bacteria within the ozonated group.

We conducted a comparative analysis of nasal tissue inflammation across groups, revealing that the control group exhibited the highest mean inflammation score. However, the differences among the groups were not statistically significant (p = 0.452). Similarly, biofilm density analysis indicated no significant differences between the groups (p = 0.248), although the control group again demonstrated the highest mean biofilm formation score. The experimental group with the lowest mean scores for biofilm formation and inflammation was Group 3 (streptomycin). Group 4 (ozone) exhibited the highest scores; these differences were not statistically significant. Chiu et al.^35^ investigated tobramycin's efficacy in preventing biofilm formation by *P. aeruginosa.* Their findings indicated that tobramycin could not eradicate the strain of *P. aeruginosa* that adhered to the mucosal surfaces. To our knowledge, this is the first experimental animal study investigating the effects of streptomycin and ozone irrigation on biofilm development in an ABRS model.

## Conclusion

This study investigated biofilm formation in an experimental ABRS model. Persistent biofilms following ABRS may contribute to recurrent disease, systemic complications, subacute infections, and in rare cases progression to chronic rhinosinusitis. In the present study, although no statistically significant differences were observed between groups in terms of inflammation and biofilm formation, irrigation procedures were associated with a reduction in bacterial load. To our knowledge, this is the first experimental animal study evaluating the effects of streptomycin and ozone irrigation on biofilm development in an ABRS model. While the streptomycin group demonstrated the lowest mean inflammation and biofilm scores, these differences were not statistically significant. Therefore, these findings should be interpreted cautiously, particularly considering the preclinical nature of the study and the relatively small sample size. Further studies with larger experimental groups and more robust quantitative biofilm assessment methods are needed to better clarify the potential role of irrigation-based approaches in the management of ABRS.

## ORCID ID

Gökhan Tüzemen: 0000-0001-9358-2019

Nazmiye Ülkü Tüzemen: 0000-0003-3544-3509

Zehra Avcı Küpeli: 0000-0003-1853-4679

Cüneyt Özakın: 0000-0001-5428-3630

## Authors' contributions

All authors contributed to the study’s conception and design. GT performed surgical and irrigation procedures. NÜT and CÖ performed microbiological procedures, and ZAK performed histopathological procedures. GT and NÜT wrote the first draft of the manuscript, and all authors commented on previous versions. Writing-review, and editing were performed by CÖ. All authors read and approved the final manuscript.

## Ethical approval

Ethical approval was obtained from the Experimental Animal Breeding and Research Unit of the Faculty of Medicine at Bursa Uludag University (Approval ID: 2022-10/04). The clinical *S. aureus* isolate used in this study was obtained with written informed consent, and ethical approval was granted by the Bursa Uludag University Faculty of Medicine Ethics Committee (no: 2022-13/21).

## Funding

This study was supported by the Scientific Research Project (THIZ-2022-1202).

## Data availability statement

The data that support the findings of this study are available from the corresponding author upon reasonable request.

## Declaration of competing interest

The authors declare no conflicts of interest.
